# Semi-Quantitative Method of Assessing the Thrombogenicity of Biomaterials Intended for Long-Term Blood Contact

**DOI:** 10.3390/ma16010038

**Published:** 2022-12-21

**Authors:** Maciej Gawlikowski, Roman Major, Barbara Mika, Dariusz Komorowski, Karolina Janiczak, Ewaryst Tkacz, Anna Tamulewicz, Natalia Piaseczna

**Affiliations:** 1 Faculty of Biomedical Engineering, Silesian University of Technology, Roosevelt Str. 40, 41-800 Zabrze, Poland; 2 Artificial Heart Laboratory, Foundation of Cardiac Surgery Development, Wolności Str. 345a, 41-800 Zabrze, Poland; 3 Institute of Metallurgy and Material Engineering, Polish Academy of Sciences, Reymont Str. 25, 30-059 Cracow, Poland

**Keywords:** thrombogenicity, biomaterials, hemocompatibility, fractal dimension

## Abstract

Biomaterials used in cardiosurgical implants and artificial valves that have long-term contact with blood pose a great challenge for researchers due to the induction of thrombogenicity. So far, the assessment of the thrombogenicity of biomaterials has been performed with the use of highly subjective descriptive methods, which has made it impossible to compare the results of various experiments. The aim of this paper was to present a new semi-quantitative method of thrombogenicity assessment based on scanning electron microscope (SEM) images of an adhered biological material deposited on the surfaces of prepared samples. The following biomaterials were used to develop the proposed method: Bionate 55D polyurethane, polyether-ether ketone, Ti6Al7Nb alloy, sintered yttria-stabilized zirconium oxide (ZrO_2_ + Y_2_O_3_), collagen-coated glass, and bacterial cellulose. The samples were prepared by incubating the biomaterials with platelet-rich plasma. In order to quantify the thrombogenic properties of the biomaterials, a *TR* parameter based on the fractal dimension was applied. The obtained results confirmed that the use of the fractal dimension enables the quantitative assessment of thrombogenicity and the proper qualification of samples in line with an expert’s judgment. The polyurethanes showed the best thrombogenic properties of the tested samples: Bionate 55D (*TR* = 0.051) and PET-DLA 65% (average *TR* = 0.711). The ceramics showed the worst thrombogenic properties (*TR* = 1.846). All the tested materials were much less thrombogenic than the positive control (*TR* = 5.639).

## 1. Introduction

Heart and circulatory system disorders are some of the most common human diseases in the 21st century [1]. Modern medical devices, such as artificial valves, heart prostheses, blood pumps, oxygenators, vascular prostheses, stents, and implantable cardioverter-defibrillators, are being increasingly applied to support therapy and treatment. Since the selection of materials with excellent biocompatibility is of particular importance for long-term implantable medical devices [2,3], researchers have developed various methods of assessing the thrombogenicity of biomaterials [4,5,6,7]. The biomaterials used for medical devices are a significant challenge for researchers because they must meet rigorous biocompatibility requirements, as detailed in the ISO 10993 standard [8]. In this paper, we focus on biomaterials’ thrombogenicity as a crucial feature in the context of cardiovascular disorders.

The set of biochemical mechanisms that keep blood in a liquid form is called hemostasis [9]. One of the several components of hemostasis is blood clotting, which prevents the blood leak from damaged blood vessels. Platelets are electrostatically attracted to the site of vessel damage, where they form an unstable platelet plug. The plug is then mechanically stabilized by polymerysing plasma-dissolved fibrinogen into insoluble fibrin. This mechanism is beneficial in damaged blood vessels but undesirable in the case of blood contact with artificial biomaterial. Biomaterials activate platelets to varying degrees, resulting in the formation of thrombi on their surface that have different sizes, but similar morphology. Platelets can be activated, among others, by excessive shear stresses generated during blood flow, by the influence of chemical factors (i.e., endogenous toxins or chemical compounds released from biomaterials), and electrically by the action of the zeta potential. Clots that form on the surface of the biomaterial are the result of the coagulation process, which is preceded by platelet activation. The thrombogenicity of the biomaterial can be assessed by measuring the percentage of activated platelets (labelled with the CD62P antibody) with flow cytometry in vitro and in vivo [10]. The marker used in the case of testing thrombogenicity caused by mechanical or chemical impact on blood is the measurement of the content of platelet microparticles; that is, the defragmented membranes of platelets. ELISA immunoenzymatic methods are used for this purpose.

Up to now, the assessment of biomaterials’ thrombogenicity has been carried out based on highly subjective descriptive methods [11]. First, the use of such approaches does not allow for comparisons of results obtained from different experiments. Second, the individual variability of blood parameters used for study has a significant impact on the results of thrombogenicity evaluation. Another factor that leads to qualitative and subjective thrombogenicity judgments is the fact that monoclonal antibodies coupled with a fluorophore are usually used for the specific labeling of the biological materials adhered to the surface of tested samples [12]. Because of the photobleaching effect [13], the parameters of obtained fluorescent images depend on the method of sample exposure (i.e., both the time and the number of exposures).

Examination of samples containing biological material with the SEM technique requires sputtering a layer of gold to avoid damaging proteins. The process is not complicated and the cost of examining one sample in a commercial laboratory is about 10 EUR per sample. Another imaging technique is scanning confocal microscopy. It requires labeling cells with fluorophore-conjugated antibodies, which makes it much more expensive than using SEM (approx. five times the cost). The use of antibodies also makes the test itself more difficult, as the sample must be in an incubator. Thus, the novel method proposed in this article, hereafter referred to as “static thrombogenicity”, could meet the expectations of material engineers as a fast, cheap, and quantitative test for the initial study of the thrombogenicity induced by biomaterials, especially in the case of polymers, metals with surface modifications, ceramics, composites, and even polymers of biological origin. This study shows that the use of the fractal dimension enables the quantitative assessment of the thrombogenicity of samples for their proper qualification into classes of material with defined levels of thrombogenicity.

## 2. Material and Methods

### 2.1. Materials

The following biomaterials were used to develop a static method of thrombogenicity assessment in the field of biological material preparation: Bionate 55D polyurethane (DSM Biomedical Inc., Netherlands), polyether-ether ketone (PEEK) (Invibio Ltd., Thornton-Cleveleys, UK), raw and TiN layer-coated Ti6Al7Nb alloys (Western Alloys, Shanghai, China), sintered yttria-stabilized zirconium oxide (ZrO_2_ + Y_2_O_3_) (Aliaxis Friatec, Mannheim, Germany), collagen-coated glass (Neuvitro Corporation, Sondheim, Germany), bacterial cellulose (Bowil Biotech, Władysławowo, Poland), and copolymers of poly(ethylene terephthalate) (PET) and dimer linoleic acid (DLA) with 65% wt. and 70% wt. of PET hard segment contents (hereinafter referred to as the abbreviations PET-DLA 65% and PET-DLA 70%, respectively) [14]. The method was developed based on the selection of the experimental conditions (such as the parameters of blood centrifugation) needed to obtain appropriate platelet-rich plasma, the duration of the experiment, and the method of securing the clots stuck to the surface of the biomaterial.

Bionate 55D is a thermoplastic polycarbonate polyurethane often used in medical applications in contact with blood [15]. Therefore, it was selected as a reference material (negative control). The Optima version of PEEK is also suitable for devices intended to come into contact with blood [16]. However, Bionate is a more modern material and better tested in terms of thrombogenicity. The collagen-coated glass was used as a positive control because collagen strongly activates platelets [17].

### 2.2. Sample Preparation

The biomaterial samples used for testing were prepared in the form of discs 5–8 mm in diameter and about 1 mm thick. The sample area could not be larger than about 30 mm^2^ because the risk of the inhomogeneous coverage of a sample surface with a biological material increases with increasing sample surface area. The samples were prepared in such a way that their surface was free from damage, chipping, and scratches. The arithmetic mean roughness coefficient (Ra) of the prepared samples was less than 0.16. Materials with a highly developed surface (e.g., coarse sinters and electrospinning materials) may not be suitable for static thrombogenicity testing because their surface structures will interfere with subsequent imaging examinations. In the presented research, polymer, metal, and ceramic samples were produced with the following methods: high-pressure injection, machining, and high-temperature sintering, respectively. The required smoothness was achieved by one-sided polishing with a diamond paste (type SD 16/10). The roughness was measured with a MarSurf M 400 (Mahr Inc., Providence, RI, USA) instrument. The samples prepared in this way were first washed in 70% ethanol with an ultrasonic cleaner, then washed in distilled water, and finally dried in a laminar chamber before being packed and sterilized with ethylene oxide (ETO).

### 2.3. Blood Donation and Platelet-Rich Plasma Preparation

Blood was collected for the anticoagulant CPDA-1 from healthy donors who had not taken anticoagulants, including acetylsalicylic acid, clopidogrel, or warfarin/acenocumarol, for at least two weeks. After collection, the blood was stored for up to 24 h at 2–7 °C. Before further tests, the following parameters were checked: blood count (BC 2800 VET hematology automaton, Mindray, Shenzhen, China), platelet aggregation under adenosine diphosphate (ADP) and arachidonic acid (multiplate impedance aggregometer, Roche, Switzerland, with ADPtest and ASPItest tests), and the concentration of plasma-free hemoglobin (fHB) (spectrophotometer Plasma/Low Hb, Hemocue AB, Ängelholm, Sweden). The following conditions were fulfilled in order to qualify blood for further tests: the blood count was normal, the platelet count was >120 × 10^3^ L/uL, the ADP test result was >122 AUC, the ASPI test result was >136 AUC, and the fHB < 0.2 g/dL.

Platelet-rich plasma (PRP) [18] was prepared immediately prior to testing via the centrifugation of whole blood at 100 G for 10 min at room temperature. The plasma morphology of the PRP was examined prior to its use in the experiment.

### 2.4. Course of Experiment

Investigated samples were placed into polypropylene tubes with a flat bottom (Falcon type) with a volume of 50 mL and a diameter of 30 mm. Each sample was placed in a separate tube in such a way that the area not subjected to final machining and polishing was on the bottom. Tubes were filled with 10 mL of PRP. The incubation of the tested samples with PRP was carried out for 60 min at a temperature of 37 °C. During the incubation, the test tubes were placed on the hematology cradle with deflection =± 5° and frequency = 10 cycles/min, which ensured the even coverage of the samples with platelets and their aggregates. At the end of incubation, samples were withdrawn from the tubes, gently washed in phosphate-buffered saline (PBS), and then fixed in 4% buffered formalin. The materials prepared in this way were sent for imaging tests.

### 2.5. Image Acquisition and Pre-Processing

**Figure 1:**
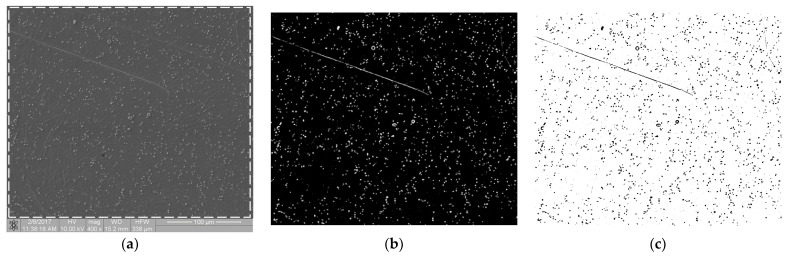
Image processing in the static thrombogenicity method for a Ti6Al7Nb + TiN sample: (**a**) raw image from SEM with a resolution of 1024 × 1024 pixels and a marked ROI (dashed line, resolution 1024 × 943 pixels), (**b**) binary image after Otsu thresholding, and (**c**) negative of binary image after Otsu thresholding (added in order to improve visibility of platelet aggregates).

The biological materials deposited to the surface of the samples were dehydrated in 70% ethanol and then covered with a gold–palladium layer in a vacuum sputtering machine. The samples prepared in this way were examined with an FIB Quanta 3D (Thermo Fisher Scientific, Waltham, MA, USA) scanning electron microscope (SEM). Images were acquired using secondary electrons under an accelerating voltage of 10.00 kV. For each sample, at randomly selected regions, at least n = 6 scans of an area of 333 × 333 µm in size at a magnification of 400× were performed. SEM scans were performed at a magnification of 400×, 800× and 1600×. The 400× magnification allowed for the imaging of individual thrombocytes over a large sample area. The remaining magnifications showed a too small fragment of the sample surface, so the cell distribution was inhomogeneous and not representative of an entire sample. Figure 1a shows an SEM scan of a sample comprising a titanium alloy covered by an anti-thrombogenic layer. In the upper left part of the image, the mechanical damage to the sample surface is visible (scratch). RGB images with a region of interest (ROI) resolution of 1024 × 943 pixels (333 × 310 µm; see Figure 1a) were obtained with the microscope. Then, the images were converted to 8-bit images and the minimum thresholding method for bimodal histograms [19], or the cross-entropy method (Otsu method) for unimodal histograms [20,21], was applied (Figure 1b). Figure 1 is available in full resolution as Supplementary Material Figure S1.

### 2.6. Calculation of Fractal Dimension

The box counting method [22,23,24] was used to calculate the fractal dimension (*D*) of images. In this method, a binarized image is placed on a regular, square grid with a side length of δ pixels. A single element of this mesh is called a box. The following set of δ parameters is most often adopted: δ = [2, 4, 6, 8, 12, 16, 32, 64] pixels. The number of boxes N(δ) that contain at least one pixel of the binarized image (so called full boxes) is then counted.

In the box-counting method, the fractal dimension *D* is calculated according to the following formula:

(1) $$D=\underset{\delta \to 0}{\lim}\frac{\log\left(N\left(\delta \right)\right)}{\log\delta }$$

In practice, linear regression is performed on a set of points with coordinates [log(*δ*), log(*N*(*δ*))]. The fractal dimension *D* is the value of the slope of the regression line.

**Figure 2:**
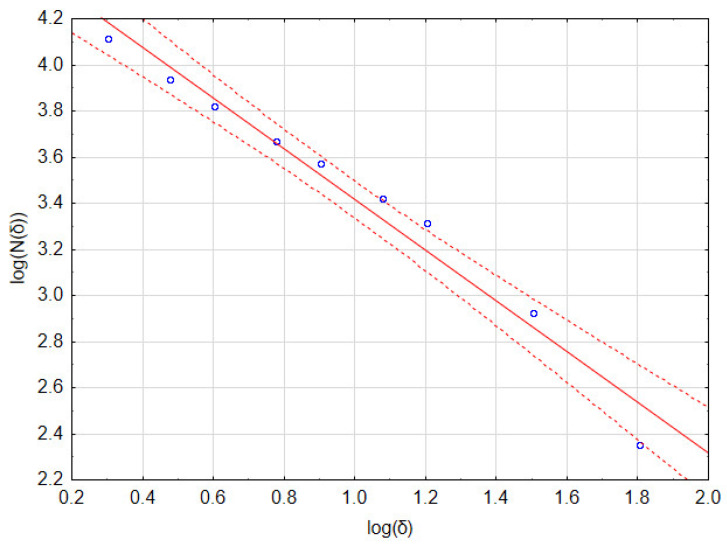
Linear regression of data from Table 1 (for Ti6Al7Nb + TiN sample, presented in Figure 1).

As an example, consider the image in Figure 1b that has a resolution of 1024 × 946 pixels. Calculations of the fractal dimension *D* were performed for the following set of *δ* parameters: 2, 3, 4, 6, 8, 12, 16, 32, and 64 pixels. In Table 1, the number of full boxes *N*(*δ*) for different box sizes δ and the decimal logarithms of these values are shown. Figure 2 and Table 2 show a graph of and detailed linear regression results for these data, respectively. It should be noted that all the regression coefficients were statistically significant at *p* = 0.05. This allows us to state that, for this image, the fractal dimension *D* (which is the slope of the regression line) equaled 1.097.

Since the range of variability of the fractal dimension *D* for the processed images of biomaterial samples dotted with thrombi was small, the raw *D* values were transformed according to Equation (2). In this way, the *TR* coefficient, which quantitatively characterizes the thrombogenic properties of a biomaterial, was calculated.

(2) $$TR=e^{D}-1$$

The use of the exponential function increased the range of variability of the raw fractal dimension *D*, and the subtraction of 1 meant that the *TR* parameter assumed a value close to 0 for samples with a very low thrombogenicity.

### 2.7. Final Statistical Analysis

The thrombogenicity of the entire sample set was expressed based on the statistical analysis of the set of *TR* parameters obtained for the samples:

- Outliers were detected with Tukey’s quartile method and removed from the statistics.
- The normality of the sample distribution was tested with the Shapiro–Wilk test at a significance level of *p* = 0.05 (a statistically significant test result indicates no normal data distribution).
- In the case of normal distribution, the thrombogenicity of each sample was quantified as the mean value of the *TR* parameters with a confidence interval of 0.95.
- In the case of an abnormal distribution, the data were analyzed with non-parametric equivalents of parametric tests: the Kruskal–Wallis’ test for the analysis of variance and the Mann–Whitney U test for the Student’s *t*-test.

## 3. Results

### 3.1. Determination of TR Parameter for Samples with Different Levels of Thrombogenicity

**Figure 3:**
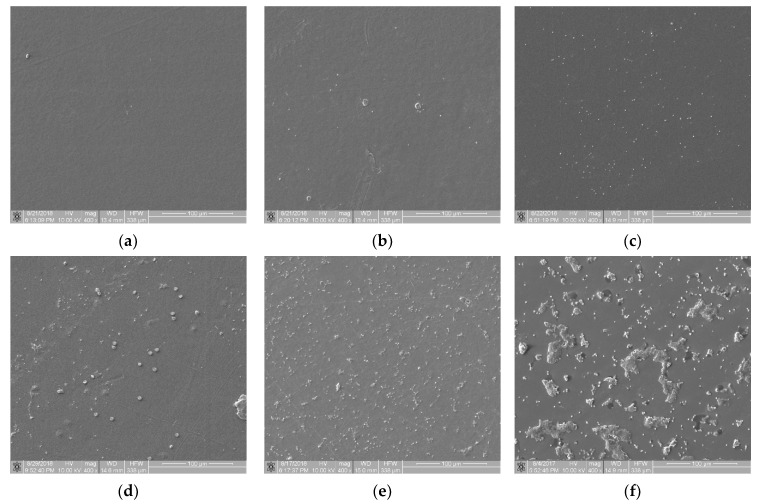
View of sample surfaces in individual thrombogenicity classes, determined on the basis of expert judgment: (**a**) class 0: minimal, *TR* = 0.051 (Bionate 55D, negative control); (**b**) class 1: very low, *TR* = 0.531 (PET-DLA 65% before biodegradation); (**c**) class 2: low, *TR* = 0.892 (PET-DLA 70% before biodegradation); (**d**) class 3: average, *TR* = 1.430 (PET-DLA 65% after biodegradation); (**e**) class 4: high, *TR* = 1.748 (PET-DLA 70% after biodegradation); (**f**) class 5: very high, *TR* = 5.639 (collagen-coated glass, positive control).

From a set of images obtained during the static thrombogenicity studies of the materials listed in Section 2.1, samples with visible mechanical damage (e.g., scratches and cracks) and other artifacts (e.g., crystallized substances and impurities) were removed. Next, n = 31 images with the homogeneous surface coverage of biological material were selected for further analysis. Based on an expert’s assessment, they were divided into six classes of thrombogenicity depending on the degree of surface coverage with plaques and their aggregates of various organization levels:

- Class 0 (n = 3) contained samples with a minimal degree of thrombogenicity: separated platelets of a low diversity level and a lack of platelet aggregates (Figure 3a);
- Class 1 (n = 3) contained samples with a very low degree of thrombogenicity: a dozen or so adhered blood platelets that did not form aggregates (Figure 3b);
- Class 2 (n = 7) contained samples with a low degree of thrombogenicity: several dozen visible platelets that could form single, separated aggregates of a small area (Figure 3c);
- Class 3 (n = 10) contained samples with an average degree of thrombogenicity: the biological material mainly comprised aggregates that showed a greater degree of thrombogenicity than that of individual platelets (Figure 3d);
- Class 4 (n = 5) contained samples with a high degree of thrombogenicity: the sample was covered with highly differentiated biological material and the individual objects were usually connected with each other, which made it impossible to separate them (Figure 3e);
- Class 5 (n = 3) contained samples with a very high degree of thrombogenicity: platelets were highly differentiated and formed numerous aggregates connected to each other, which made it impossible to separate and count objects (Figure 3f).

**Figure 4:**
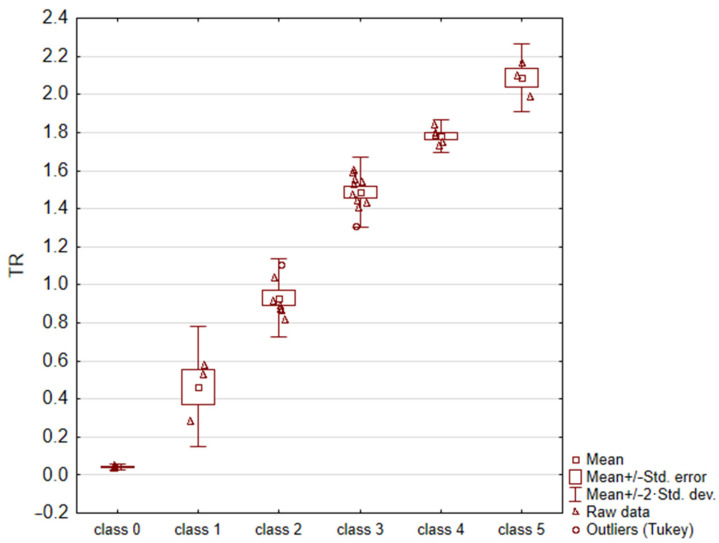
Results of statistical analysis (ANOVA test with Welch’s correction).

For each image, the fractal dimension *D* and the *TR* = *e^D^* − 1 parameter were calculated. Due to the non-homogeneity of variance (result of C-Cochran’s test *p* = 0.0466), an analysis of variance with Welch’s correction (*p* = 0.000), and Bonferroni’s post-hoc test (for each combination, the test result was statistically significant: *p* < 0.010) were applied to the data. The obtained results are summarized in Table 3 and Figure 4. According to these results, it can be concluded that the *TR* parameter enabled the unambiguous assignment of a sample surface image to one of the six thrombogenicity classes.

### 3.2. The Influence of the Unique Features of the Image on the Value of the TR Coefficient

In order to establish the link between unique feature of the image and *TR* parameter value, three types of potentially unfavorable features in analyzed images were specified. The inhomogeneous coverage of the sample with biological material, artifacts on the surface of the sample (e.g., crystallized chemical compounds), and mechanical damage to the surface of the sample were taken into account. Following this, their influence on fractal dimension *D* and the value of the *TR* coefficient were examined.

**Figure 5:**
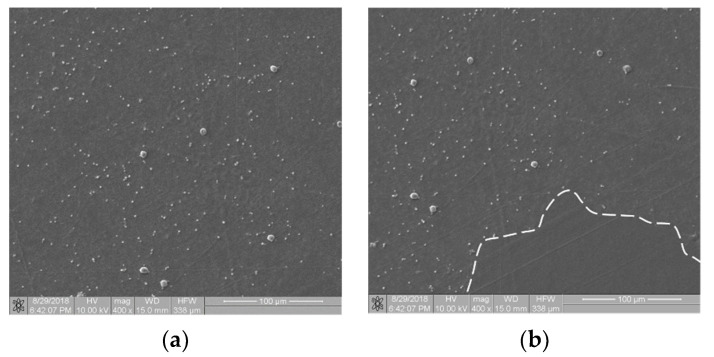
Thrombogenicity of Ti6Al7Nb + TiN material: (**a**) image of an area evenly covered with platelets; (**b**) image of an area unevenly covered with platelets (there are no visible platelets in the region marked by dashed line).

Referring to the inhomogeneous coverage of a sample with biological material, Figure 5 presents two images from different places on the same sample (Ti6Al7Nb + TiN coating): with even (Figure 5a) and uneven (Figure 5b) coverage with biological material. The fractal dimension *D* and *TR* coefficient values were as follows: *D*(a) = 0.831 and *TR*(a) = 1.307 and *D*(b) = 0.8231 and *TR*(b) = 1.277. They differed from each other by about 4.5%. This observation allows us to hypothesize that the *TR* parameter is resistant to uneven sample coverage. We intend to confirm this premise in further research.

**Figure 6:**
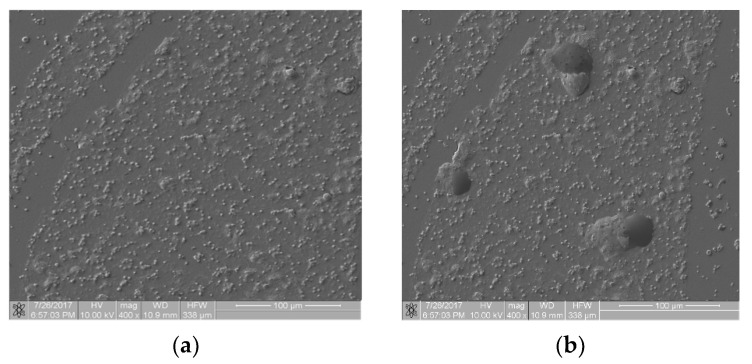
Thrombogenicity of ZrO_2_ + Y_2_O_3_ material: (**a**) image of an area free from artifacts; (**b**) image of an area including three artifacts.

Figure 6 presents images from the static thrombogenicity test of a sample comprising ZrO_2_ + Y_2_O_3_: image (a) is free from artifacts and image (b) shows three artifacts of unknown chemical composition. The fractal dimension *D* and *TR* coefficient values of the images were: *D*_(a)_ = 1.046, *TR*_(a)_ = 1.846, *D*_(b)_ = 1.211, and *TR*_(b)_ = 2.356. They differed from each other by approximately 28%. These preliminary results suggest caution in the case of artifacts in the analyzed images because they affected the value of the *TR* coefficient. As the values of *TR* parameters differed by more than 25%, at this stage of the research, this difference cannot be ignored. This problem will be the subject of further analysis.

**Figure 7:**
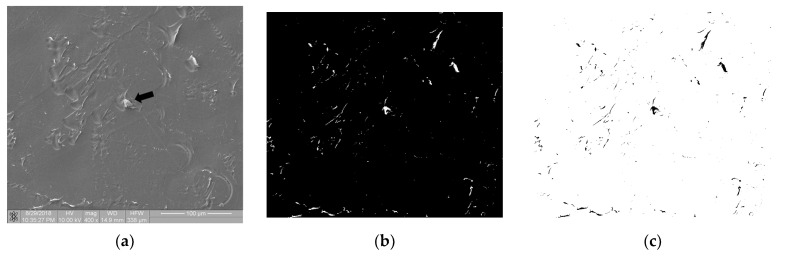
Thrombogenicity of PEEK material: (**a**) image of the sample surface with an adhered single-platelet aggregate indicated with black arrow; (**b**) image after thresholding containing numerous erroneous highlighted areas; (**c**) negative of the binary image after thresholding.

Figure 7 shows the image of surface of the polymer sample (PEEK) after the static thrombogenicity test. The visual assessment of the sample surface (Figure 7a) focused on the presence of one platelet aggregate (labeled with a black arrow) and numerous signs of mechanical damage. After thresholding using the maximum entropy method, the binarized image was found to contain areas that were highlighted even though they were not biological material. The fractal dimension *D* and *TR* coefficient of this image were 0.835 and 1.304, respectively. According to the class of thrombogenicity division presented in Table 3, such a sample with a minimum degree of coverage should have been assigned to class 0 with a *TR* parameter value of 0.05, which is inconsistent with the obtained results.

The performed research confirmed that the mechanical damage of the sample surface may completely distort its thrombogenicity assessment by means of the described method, so such cases should be excluded from analysis earlier. Figure 7 is available in full resolution as Supplementary Material Figure S2.

### 3.3. Boundary Cases

Two boundary cases of samples with a mechanically undamaged surface are considered below: a case where very few platelets adhered to the surface of the test sample and a case where the sample was heavily covered with biological material.

**Figure 8:**
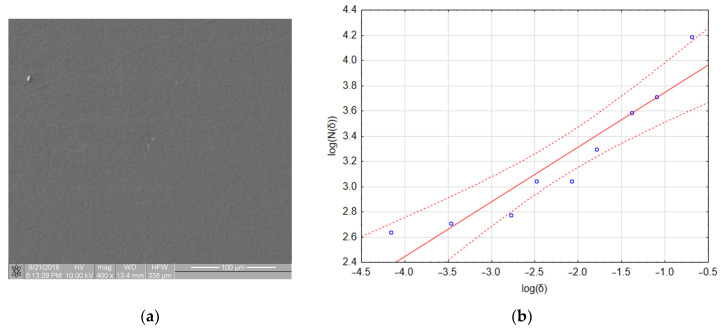
Boundary case: (**a**) sample with a very small number of adhered platelets; (**b**) calculation of fractal box count—poorly fitted regression model.

Figure 8a shows an image of a sample with a very small amount of adhered biological material. One aggregate and two platelets are visible in the image. However, the fractal dimension of this image was 0.4327 and its *TR* coefficient was 0.541, which places this material in class 1 (refer to Table 4). The linear regression assessment (Figure 8b) showed that the quality of the regression model was poor (refer to Table 4 and notice that three out of nine points lie outside the confidence interval of the model). However, if the regression only included data obtained for boxes with sizes of 64, 32 and 16 pixels (the first three points on the left side of the graph), the fractal dimension dropped to a value of 0.093 and the *TR* value dropped to 0.097, which placed this material in class 0. An explanation for this effect and its consequences for the presented method of thrombogenicity assessment are presented in the discussion section.

**Figure 9:**
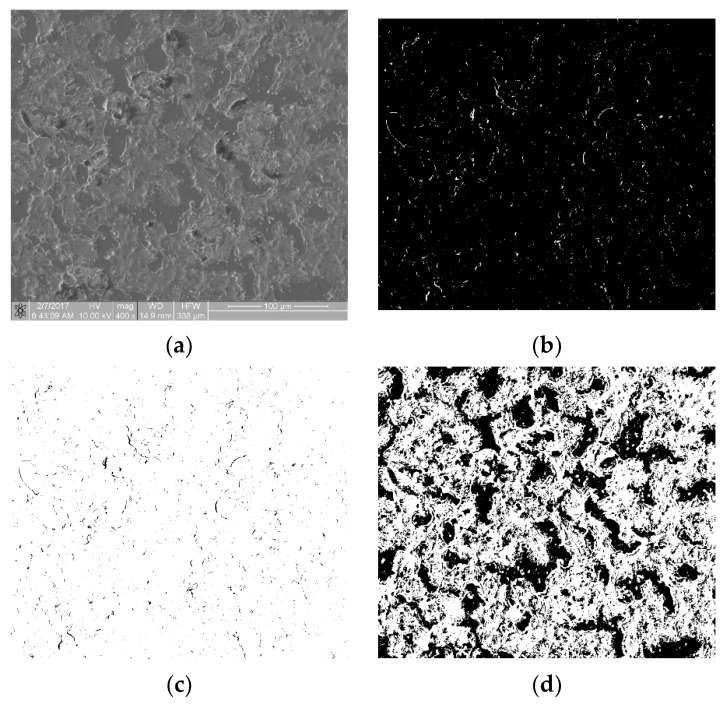
Boundary case: (**a**) sample with very high thrombogenicity; (**b**) max entropy method thresholding; (**c**) negative of binary image after max entropy method thresholding; and (**d**) intermodal method thresholding.

Figure 9a shows a sample with a very high degree of coverage with biological material. In this case, the proper qualification process depended on the thresholding method. Thresholding using the max entropy method yielded the image shown in Figure 9b. Its fractal dimension *D* was 0.871 and its *TR* was 1.389, which completely erroneously places this sample between thrombogenicity classes 2 and 3. The histogram of this image was found to be bimodal. The best results of thresholding for such an image were obtained with the intermodal method [19] (Figure 9c). The obtained fractal dimension *D* = 1.900 and the *TR* coefficient = 5.685, correctly placing the image (Figure 9c) in class 6 for samples of a very strong thrombogenicity. In conclusion, in order to obtain the correct values of the *D* and *TR* parameters, images with a bimodal histogram should be binarized using the intermodal method. Figure 9 is available in full resolution as Supplementary Material Figure S3.

## 4. Discussion and Conclusions

The proposed method based on the fractal dimension and the *TR* parameter for the qualification of a sample surface to individual thrombogenicity classes seems to be promising. The performed statistical analysis confirmed that, in most of the studied cases, the *TR* parameter enabled an unambiguous assignment of the sample surface image to one of the six thrombogenicity classes, although there were some special sample cases that required another approach.

For example, when there was a lack of bright pixels in the image after thresholding, the fractal dimension did not exist because of *N*(*δ*) = 0, which means that log(*N*(*δ*)) also did not exist. In such a case, the *TR* parameter should be assumed to be zero. According to the authors’ research, the proposed *TR* parameter used for classification is not only sensitive to both the artifacts on the sample surface and their mechanical damage but also robust to the inhomogeneous sample coverage of biological materials.

In some boundary cases, described in detail in Section 3.3, there were problems in the assignment of the image to the proper class of thrombogenicity, mainly because of the poor quality of the linear regression model for the determination of the fractal dimension. In these instances (e.g., Figure 8a), interval regression should have been used and the fractal dimension obtained for large boxes presented in an area of image elements, whereas the fractal dimension determined for small boxes could be treated as the degree of structure complexity. In images such as that in Figure 8a, the fractal dimension *D* obtained for boxes of 64, 32, and 16 pixels was equal to 0.093; in contrast, the fractal dimension *D* obtained for small boxes of 8, 6, 4, 3, and 2 pixels was equal to 0.6513, and while the whole image it was *D* = 0.4327. Thus, when determining the fractal dimension of an image with an extremely low degree of coverage with biological material (e.g., Figure 8a), the authors recommend assessing the input data for the regression process and separately using interval regression for large and small boxes instead of linear regression. The performed studies confirmed that for such images, the fractal dimension presenting the degree of surface thrombogenicity provides better results for big boxes. Similar conclusions were obtained by other investigators [25], who analyzed some cases where linear regression did not unequivocally describe the fractal dimension.

It seems worthy to compare the quality of the performed classification to outcomes obtained for the other methods used for fractal dimension determination. Therefore, in the future, we would like to consider the results of the presented thrombogenicity classification by using a box-counting method and a mass-radius method for the fractal dimension calculation. Using only one method to analyze the complexity of objects may be insufficient. It can be presumed that a combination of both methods for fractal dimension calculation can support the image classification process. It may be assumed that the features of an examined object expressing the complexity of its outline shown in a two-dimensional graph with axes referring to the fractal dimension estimated with the box-counting and mass-radius methods typically cause objects to have a clear tendency to be grouped into clusters. In this paper, we present the preliminary step for surface thrombogenicity classification.

Of the tested samples, the following polyurethanes had the best thrombogenic properties: Bionate 55D (*TR* = 0.051) and PET-DLA 65% (average *TR* = 0.711). In the case of PET-DLA, the material’s thrombogenicity deteriorated after six months of biodegradation in SBF (simulated body fluid). Bionate is a polyester-based polyurethane that is often used in medicine to construct devices that come into prolonged contact with blood, such as blood pumps, dialyzers, cannulas, and catheters. Similar properties were demonstrated with a titanium alloy (Ti6Al7Nb) with a TiN anticoagulation layer (*TR* = 0.831). Other polymers such as PEEK were shown to have a higher thrombogenicity (*TR* = 1.304). The thrombogenicity of the ceramic samples (ZrO_2_ + Y_2_O_3_) was *TR* = 1.846. All the mentioned materials were significantly less thrombogenic than the positive control, for which *TR* = 5.639.

## Figures and Tables

**Table 1:** Raw results of the box method of image fractal dimension calculation.

Box Size δ (Pixels)	2	3	4	6	8	12	16	32	64
*N*(*δ*)	13,011	8666	6606	4642	3737	2639	2072	844	224
log(*δ*)	0.301	0.477	0.602	0.778	0.903	1.079	1.204	1.505	1.806
log(*N*(*δ*))	4.114	3.938	3.820	3.667	3.573	3.421	3.316	2.926	2.350

**Table 2:** Results of linear regression of data from Table 1 and fractal dimension assessment.

Parameter	Value	Standard Error	*p*
intercept	4.513	0.180	0.000
slope	−1.097	0.073	0.000
R^2^	0.984	0.9698	0.001
*D* = ABS(slope)	1.097	0.073	0.000
*TR_N_* = *e^D^* − 1	1.995	−0.227/+0.211	

**Table 3:** The average values of the *TR* coefficient for the analyzed images in each group.

Class of Thrombogenicity	Sample Size n	Mean *TR*	Upper Confidence Interval	Lower Confidence Interval
class 0	3	0.043	0.024	0.061
class 1	3	0.465	0.071	0.858
class 2	7	0.930	0.835	1.026
class 3	10	1.488	1.422	1.554
class 4	5	1.783	1.729	1.837
class 5	3	2.087	1.867	2.307

**Table 4:** Results of linear regression obtained for the fractal dimension calculation of the image presented in Figure 7.

Parameter	Value	Standard Error	*p*
Intercept	1.479	0.153	0.000
Slope	0.432	0.062	0.000
R^2^	0.854	0.199	0.000
*D* = ABS(slope)	0.432	0.062	0.000
*TR_N_* = *e^D^* − 1	0.541	−0.098/+0.093	

## Data Availability

The data presented in this study are available on request from the corresponding author. The raw/processed data required to reproduce these findings cannot be made publicly available at this time as the data also form part of an ongoing study.

## Supplementary Materials

The following supporting information can be downloaded at: https://www.mdpi.com/article/10.3390/ma16010038/s1. Figure S1: Full resolution images–image processing in the static thrombogenicity method for a Ti6Al7Nb + TiN sample: (a) raw image from SEM with a resolution of 1024 × 1024 pixels and a marked ROI (dashed line, resolution 1024 × 943 pixels), (b) binary image after Otsu thresholding. Figure S2: Full resolution images–thrombogenicity of PEEK material: (a) image of the sample surface with an adhered single-platelet aggregate; (b) image after thresholding containing numerous erroneous highlighted areas. Figure S3: Full resolution images–boundary case: (a) sample with very high thrombogenicity; (b) max entropy method thresholding; and (c) intermodal method thresholding.
