# Supplementary figures and images for: Semi-Quantitative Method of Assessing the Thrombogenicity of Biomaterials Intended for Long-Term Blood Contact
Source: Materials (Basel). 2022 Dec 21;16(1):38. doi: 10.3390/ma16010038 (PMC9821258; doi:10.3390/ma16010038)

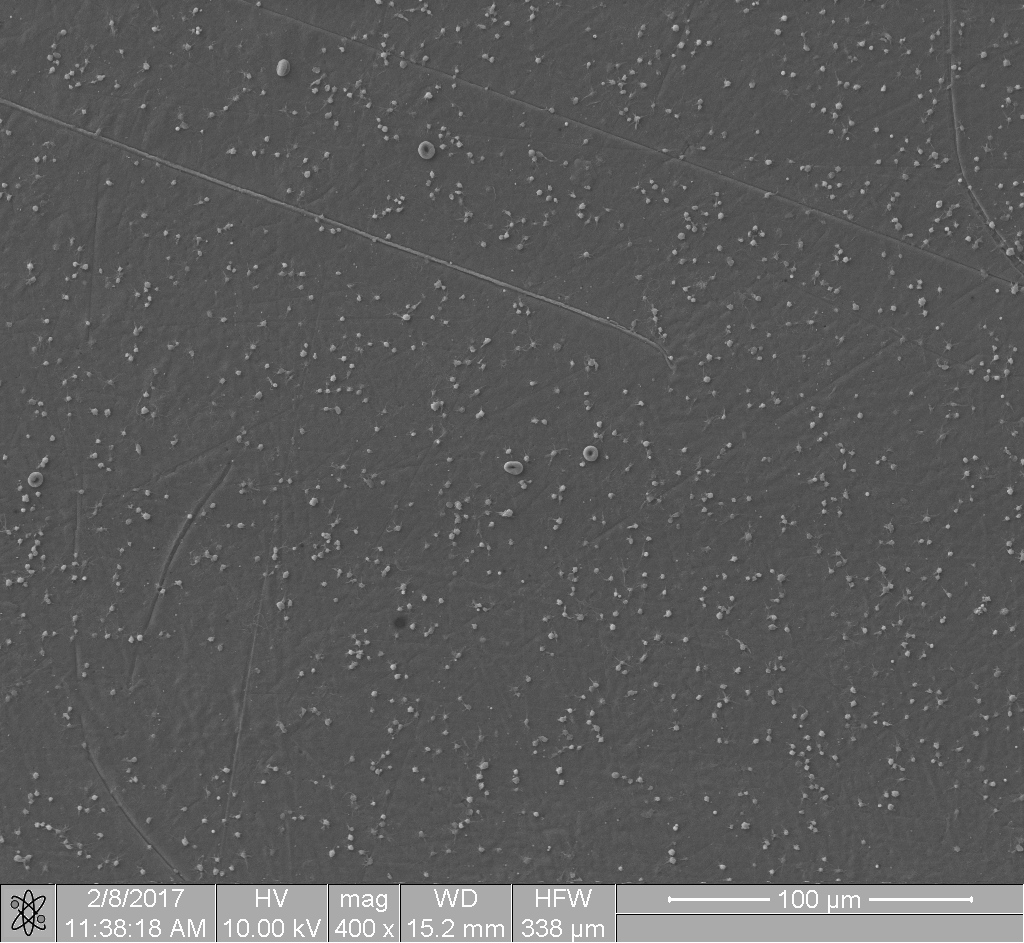

Supplement: Supplementary file 1 [file materials-16-00038-s001.zip › Figure S1a.tif]

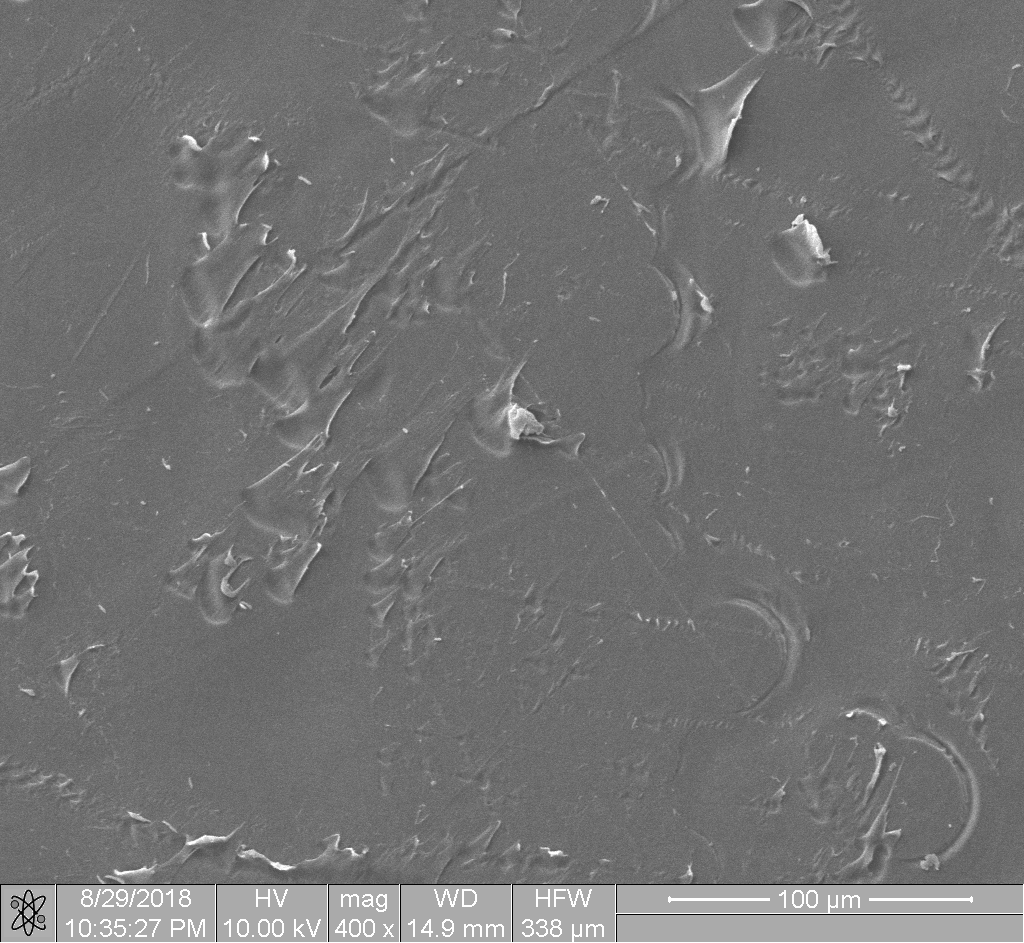

Supplement: Supplementary file 1 [file materials-16-00038-s001.zip › Figure S2a.tif]

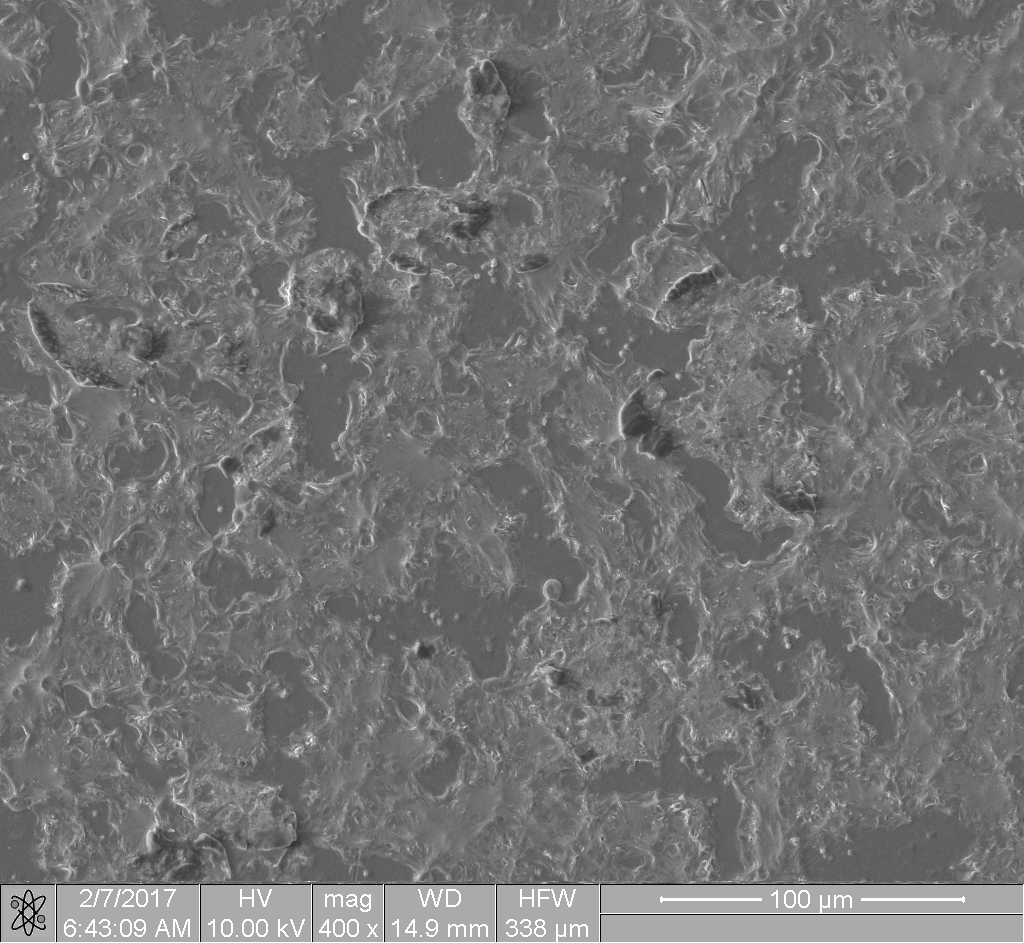

Supplement: Supplementary file 1 [file materials-16-00038-s001.zip › Figure S3a.tif]
